# The *Bacillus anthracis* Cell Envelope: Composition, Physiological Role, and Clinical Relevance

**DOI:** 10.3390/microorganisms8121864

**Published:** 2020-11-26

**Authors:** Alice Chateau, Sander E. Van der Verren, Han Remaut, Antonella Fioravanti

**Affiliations:** 1 Avignon Université, INRAE, UMR SQPOV, F-84914 Avignon, France; alice.chateau-huyot@univ-avignon.fr; 2 Structural and Molecular Microbiology, Structural Biology Research Center, VIB, 1050 Brussels, Belgium; Sander.Egbert.Van.Der.Verren@vub.be (S.E.V.d.V.); han.remaut@vub.be (H.R.); 3 Structural Biology Brussels, Vrije Universiteit Brussel, 1050 Brussels, Belgium

**Keywords:** *Bacillus anthracis*, cell envelope, S-layer, secondary cell-wall polysaccharide, peptidoglycan, S-layer-associated proteins, BSLs, capsule, phages, nanobodies

## Abstract

Anthrax is a highly resilient and deadly disease caused by the spore-forming bacterial pathogen *Bacillus anthracis*. The bacterium presents a complex and dynamic composition of its cell envelope, which changes in response to developmental and environmental conditions and host-dependent signals. Because of their easy to access extracellular locations, *B. anthracis* cell envelope components represent interesting targets for the identification and development of novel therapeutic and vaccine strategies. This review will focus on the novel insights regarding the composition, physiological role, and clinical relevance of *B. anthracis* cell envelope components.

## 1. Introduction

*Bacillus anthracis* is a Gram-positive spore-forming bacterium that exists in two morphologically and physiologically distinct states: a dormant spore and the actively growing vegetative cell. The bacterium is the etiological agent of anthrax disease, an often fatal acute disease that commonly affects livestock and wildlife animals, and more rarely, humans, across the world. Naturally occurring anthrax infections are primarily found in herbivores, which become infected by the ingestion of spores found in soil or on feed. Spores shed from carcasses of anthrax-infected animals are a primary route for infection of carnivores and form a long-lasting contamination of soils that in turn infect herbivores feeding on these grounds, even decades later [1]. Wildlife anthrax outbreaks remain endemic in many arid grass lands, steppes, and tundras across the world and have more recently also been identified as an important driver for wildlife mortality in tropical forests [2,3,4].

Interestingly, it has been brought to light that depending on environmental condition, such as arid savannahs or rainforest ecosystems, wildlife anthrax can be caused, respectively, by *B. anthracis* or by the closely related bacterium *B. cereus biovar anthracis* (Bcbva). The latter combines the chromosomal background of *B. cereus* with the toxin-encoding pXO1 and the capsule-encoding pXO2 plasmids of *B. anthracis* [5]. In contrast to *B. anthracis*, which is distributed globally [4], Bcbva causes “sylvatic anthrax”, a prevalent and persistent cause of death of a broad range of mammalian hosts in the rainforest ecosystem located in West and Central Africa regions [2,6], as well as in North American areas such as Texas and Louisiana [7].

In humans, there are three main routes of anthrax infection: cutaneous, respiratory, and gastrointestinal. Cutaneous anthrax forms the most common manifestation of human *B. anthracis* infection, representing >95% of cases. It is most often contracted by spores entering through skin cuts in individuals handling infected animal products including meat, wool, hides and leather, and more rarely, through insect bites. Cutaneous anthrax initially shows a milder manifestation, as localized blisters and skin sores, and can usually be successfully treated with antibiotics. When left untreated, however, cutaneous anthrax progresses to a fatal systemic disease in as much as 20% of cases. Although rarer as infection routes, respiratory and intestinal anthrax readily develop into systemic and rapidly deteriorating disease that is often fatal within 2 to 3 days. These anthrax forms are extremely difficult to overcome if not treated promptly and exhaustively [8]. Without treatment, the mortality rate for inhalational anthrax is as high as 90 to 100%, and even with antibiotics, mortality from inhalational anthrax in the 2001 anthrax letter cases was 45% [9]. Antibiotics such as ciprofloxacin, doxycycline, or levofloxacin represent the standard treatment for anthrax [9]. Although these antimicrobials can effectively eliminate bacteremia, anthrax is a toxin-mediated disease, and toxin accumulation is associated with mortality. To circumvent this problem, antibiotic therapy is supplemented with antibody-based antitoxin treatments [10,11].

Fortunately, with good sanitary measures and food hygiene practices, intestinal and inhalational anthrax infections are rare [4]. However, the high mortality of inhalational anthrax combined with the high persistence of spores in the environment have made this bacterium a biological agent that was once developed a as potent bioweapon [12,13]. Although the 1975 Biological Weapons Convention prohibits the use, development, and stockpiling of biological and toxin weapons by signatory nations, there is a remaining concern that weaponized anthrax spores may be used in human conflict or terrorism acts. The highly persistent and easy dissemination of *B. anthracis* spores, the high mortality and lack of an adequate treatment for acute anthrax disease, the fact that spores are relatively easy to be found in the environment, and that they require a limited knowledge to use them to harm people have made this bacterium a renowned dreadful bio-treat that can cause collective panic in cases of intentional release. As such, *B. anthracis* is in fact classified as a Category A priority pathogen, a bacterium that poses the highest risk to the public and national security, by the Center for Disease Control and Prevention (CDC) (https://emergency.cdc.gov/agent/agentlist-category.asp). The Americans (BioThrax^®^) and the British (AVP) developed anthrax vaccines that are available and licensed for human use for military personnel and high-risk professionals [14]. Both vaccines are made from culture filtrate of a non-encapsulated attenuated *B. anthracis* strain, but they can trigger undesirable side effects, and also, the protective efficiency of these vaccines is unclear at present. Characterization of these vaccines has shown that their anthrax neutralizing activity is in large part attributed to a secreted bacterial protein, the protective antigen (PA) [15]. PA is the channel forming component required for host cell binding and delivery of the anthrax lethal and edema toxins [16]. In case of biological terrorism or warfare anthrax release, post-exposure prophylaxis (PEP) includes the use of anthrax vaccines too (CDC, ACIP summary report; October 22–23, 200). Where antimicrobials post-exposure treatment can reduce the incidence or progression of the disease, it cannot protect against the subsequent disease that might occur in case residual spores would germinate after the cessation of the recommended antibiotic regime. In this context, the AV7909 vaccine, composed by the BioThrax® vaccine in combination with the immunostimulatory oligodeoxynucleotide compound CPG 7909, was proven to enhance both the magnitude and the kinetics of antibody responses in animals and human subjects, thus making the AV7909 a suitable next-generation vaccine for use in a PEP setting [17]. Other reports also indicate a protective contribution from *B. anthracis* cell envelope components such as the S-layer proteins that cover the vegetative cells [18]. Recent reports have further indicated that *B. anthracis* S-layer formation may be a target for antibacterial therapeutics [19].

In this review, we will provide a comprehensive overview of the current understanding of the complex *B. anthracis* cell envelope composition and its physiological role, with a special focus on the latest studies reporting promising evidences that point out its potential as a target for the development of better defined vaccines as well as direct antibacterial therapies.

### Bacillus anthracis Cell Envelope

**Figure 1:**
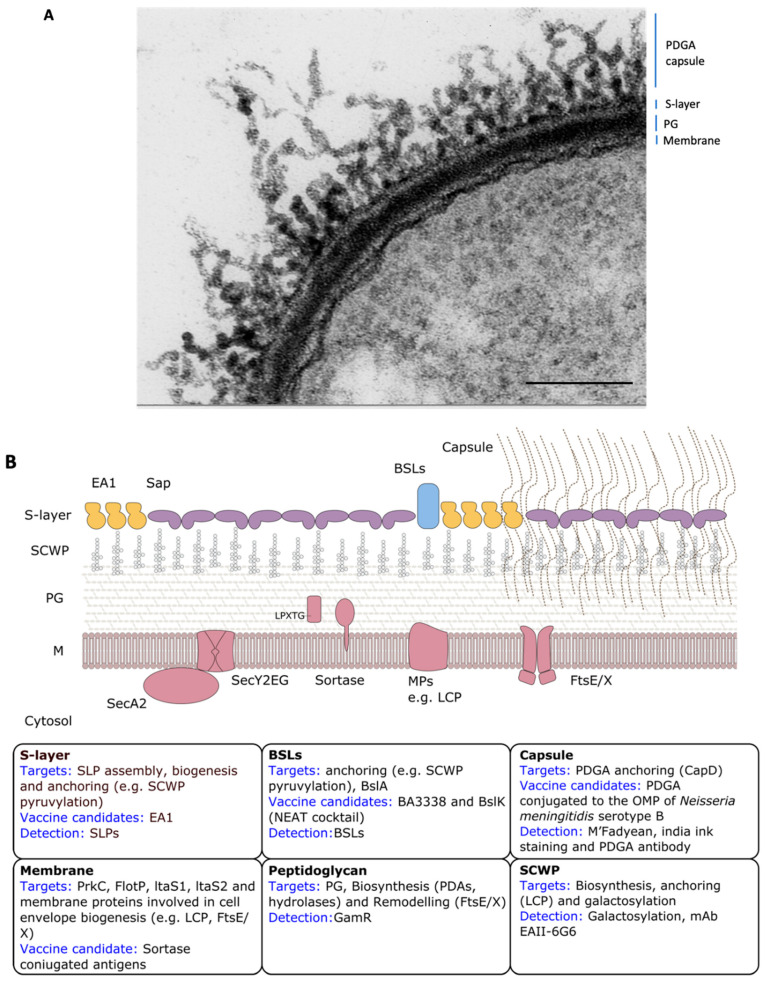
*B. anthracis* cell envelope organization. (**A**) Electron micrograph of a negative stained capsulated *B. anthracis* thin section. Adapted from Mesnage et al., 1998 [20]. Scale bar is 250 nm. (**B**) Schematic representation of cell envelope organization in presence or absence of poly-γ-d-glutamic acid (PDGA) capsule (right and left, respectively). Therapeutic targets, vaccine candidates, and detection targets are reported for each cell envelope component in the respective insets. Cell-wall components are indicated as such for both panels: cytosolic membrane (M), peptidoglycan layer (PG), secondary cell-wall polysaccharides (SCWP), surface layer (S-layer), *Bacillus anthracis* S-layer-associated proteins (BSLs), and capsule.

Cell envelopes are the vital cell boundaries between a cell and its immediate environment and provide the first line of protection against external threats. They play important roles in many essential aspects of cellular life, yet are far from being completely understood. For pathogenic bacteria, cell surfaces are at the front of the interaction with the host. The essential and readily accessible nature of cell envelope components makes them interesting targets for the development of novel antimicrobial therapies and vaccines. Therapeutics that target cell envelope components may be expected to have increased bioavailability and can circumvent resistance mechanisms such as drug efflux. *B. anthracis* possesses a complex architecture and dynamic composition of its cell envelope (Figure 1A). This includes a thick peptidoglycan (PG) layer, an associated secondary cell-wall polysaccharide (SCWP), one of two distinct proteinaceous paracrystalline arrays known as S-layers, and a poorly immunogenic and antiphagocytic poly-γ-d-glutamic acid (PDGA) capsule.

## 2. The Boundary that Enables Life: The Cytoplasmic Membrane

Cytoplasmic membranes represent the most essential and primary cell boundary that enables life. Biological membranes are composed of a lipid bilayer matrix with embedded and associated proteins. The selective permeability of membranes regulates which substances enter or leave the cells and forms the primary signaling medium with the extracellular environment. The phospholipids in the *B. anthracis* cytoplasmic membrane have been reported to slightly differ in fatty acid composition compared to those found in *B. thuringiensis* and *B. cereus* [21,22], two closely related bacteria in the *B. cereus* sensu lato group. In particular, *B. anthracis* produces a smaller proportion of three nine-branched fatty acids, the i13:0, a13:0, and i14:0 [23]. This difference was proposed as a possible discriminating criterion for strain differentiation amongst these very closely related species in case of anthrax outbreak. However, because membrane content identification is technically more challenging than DNA-based differentiation approaches, it has not been used in routine practice. Until recently, the *B. anthracis* membrane was thought to lack membrane-associated polysaccharides such as the lipoteichoic acids (LTA) that are commonly found to decorate membranes in Gram-positive bacteria. Although glycerol phosphate polymers have not been isolated from *B. anthracis* [24], there is an intact *dltABCD* operon in the genome of the bacterium [25]. In *Lactobacilus casei*, DltABCD catalyze the D-alanyl esterification of polyglycerol and polyribitol phosphates in its LTAs and wall techoic acids (WTAs) [26], leaving the possibility that these molecules could also be produced in *B. anthracis*. In 2012, Garufi and co-workers reported that *B. anthracis* indeed synthesizes LTAs and identified the four genes involved in its synthesis as *ltaS1, ltaS2, ltaS3*, and *ltaS4* [27]. Bacteria lacking both *ltaS1* and *ltaS2* were unable to synthesize LTA and exhibited reduced viability, altered envelope morphology, aberrant separation of vegetative forms, and decreased sporulation efficiency [27] (Figure 1B).

### Membrane Proteins

Membrane proteins (MPs) are involved in several functions that are crucial to bacterial life, such as selective in and out trafficking of proteins and solutes, as well as signal transduction. Because of the important role that these proteins play, they represent potential targets for the development of vaccines and novel drugs. In recent years, several research groups have performed proteomic studies in order to identify novel MPs expressed during germination and early vegetative growth stages of *B. anthracis* infection [28,29]. Tremendous work has been done by the Schneewind–Missiakas lab in recent decades to identify and characterize novel membrane proteins involved in cell-wall modification that show chain length defects when knocked out and that will be discussed in detail in the next peptidoglycan paragraph. Among them, sortases (Srts) are an important class of MPs. The genome of *B. anthracis* encodes class A, B, and D sortases, known as SrtA, SrtB, and SrtC, respectively [30]. SrtA anchors seven proteins to the cell wall by joining the threonine of the LP*X*TG sorting signal (see below) present at the C-terminus of the target protein to the amine group of *meso*-diaminopimelic acid within lipid II [31]. SrtB anchors IsdC, which is important for heme binding [32], whereas SrtC attaches two proteins involved in sporulation (BasH and BasI) [33]. The ability of *B. anthracis* to thrive and replicate within macrophages has been described as being dependent upon the display of bacterial surface proteins that are attached to the cell wall by the SrtA enzyme [34]. Two studies demonstrated that a *B. anthracis* mutant strain lacking SrtB is defective in haem-iron scavenging [35] and exhibited a decreased ability to grow intracellularly as compared with the parental wild-type Sterne strain (toxins producing pXO1^+^; non-capsulated pXO2^−^) [34]. It was thus suggested that SrtB is essential for the ability of *B. anthracis* to grow in macrophages and may be critical in the early stages of infection. The structure of *B. anthracis *SrtA has been shown to differ from that of other SrtA [36,37]. Understanding the mechanism through which this enzyme displays surface proteins is of fundamental importance and could facilitate the design of new anti-infective agents that would impair surface protein display. However, no significant difference was observed in the LD_50_s determined on mice infected by the sub-cutaneous route of each single and the triple sortase mutant both in toxinogenic non-encapsulated (pXO1^+^; pXO2^−^) and in non-toxinogenic encapsulated (pXO1^−^; pXO2^+^) backgrounds [38]. Furthermore, no difference in virulence was observed between the fully virulent Ames strain (pXO1^+^; pXO2^+^) and its *srtC* derived mutant in a guinea pig subcutaneous model of anthrax [39]. A deletion mutant of *isdC,* on the other hand, is as virulent and pathogenic to guinea pigs as the fully virulent wild-type Vollum strain [40]. These last reports suggest that targeting *B. anthracis* Srts is not the best way to combat anthrax. However, using Srt enzymes to conjugate a recombinant carrier protein with a C-terminal LPXTG motif can be useful for the development of vaccine against anthrax. For example, SrtA was used to link the PDGA capsule of *B. anthracis* to the receptor binding domain (D4) of protective antigen (PA encoded by *pagA* gene). When used as a vaccine, PDGA-D4 conjugate elicited robust antibody responses against both capsule and D4 and immunization with PDGA-D4-afforded guinea pigs complete protection against anthrax challenge with wild-type or *pagA* mutant *B. anthracis* Ames (Figure 1B) [41].

In 2016, Somani and co-workers investigated for the first time the existence of raft-like markers in pathogenic bacteria [42]. Lipid rafts are dynamic, nanoscale assemblies of specific proteins and lipids, distributed heterogeneously on cytoplasmic membrane, which coordinate membrane signaling and trafficking involving raft-associated proteins [43]. Furthermore, impairment of the lipid raft is associated with several human diseases [44,45]. In this study, the *B. anthracis* FlotP, an homologue of conserved eukaryotic raft marker protein Flotillin-1 [43], was identified and characterized. FlotP is constitutively expressed in *B. anthracis.* This suggests its potential role in various physiological and cellular processes, which might range from basic bacterial metabolism to virulence via regulatory or signaling pathways. Interestingly, treating *B. anthracis* cells with Zaragozic acid (ZA), a raft-associated lipid biosynthesis inhibitor, considerably affected their growth, morphology, membrane fluidity, and toxin secretion [42]. These evidences not only support the existence of a raft like entities in *B. anthracis* but also the role that they play in its pathology, suggesting their possible use for the development of novel drugs or vaccines against anthrax (Figure 1B). An interesting study from 2020 has identified the first sensory molecule regulating the chaining phenotype of *B. anthracis*, the PrkC serine/threonine protein kinase [46]. Such phenotype has been shown to contribute significantly to the virulence of this bacterium [47,48,49]. Previously, the disruption of PrkC in a non-capsulated *B. anthracis* strain showed decreased virulence in a mice model of pulmonary anthrax [50]. Dashmana and co-workers proposed that PrkC, perceiving growth-permissive signals, maintains the levels of proteins involved in de-chaining and cell division such as Sap, the murine hydrolase BslO, and the cytoskeletal protein FtsZ [51,52] and thus regulates the chaining phenotype. For the *B. anthracis* Sterne 34F2 *prkC* mutant, they could in fact observe an upregulation of Sap, BslO, and FtsZ together to an inability of this mutant to undergo into chaining phenotype. These latest results reinforce the potential relevance of investigating a therapeutic intervention against PrkC that could help in controlling bacterial chain size and hence the lung tissue injury and its pathophysiological consequences (Figure 1B).

## 3. The Bacterial Great Wall: The Peptidoglycan Layer

One of the best characterized and most studied components of the bacterial cell envelope, in both Gram-positive and Gram-negative bacteria, is the peptidoglycan (PG) sacculus. PG primarily functions as a stress-bearing layer to resist the internal osmotic pressure of the cytoplasm. A secondary, but equally important, role of the sacculus is to serve as a semi-rigid scaffold for attachment of other cell-wall components, such as polysaccharides and proteins.

*B. anthracis* PG is of the A1 *γ* type [53]. The glycan chain consists of alternating units of *N*-acetylglucosamine (GlcNAc) and *N*-acetylmuramic acid (MurNAc) held together by *β*1→4 glycosidic linkages, while the stem peptide is constituted of L-Ala, D-Glu, meso-diaminopimelic acid (DAP), D-Ala, D-Ala. The peptides are cross-linked by a direct linkage between the meso-DAP and the D-Ala in position 4. The structure of the peptidoglycan of a *B. cereus* strain, a close relative to *B. anthracis*, displays distinguishing structures, which are also found in *B. anthracis* peptidoglycan [53]. Many pathogens modify their peptidoglycan to resist host lysozyme [54]. Common modifications of peptidoglycan include *N*-deacetylation, *N*-glycolylation, and O-acetylation [55], but *B. anthracis* PG is not O-acetylated [56]. Deacetylated amino sugars are formed from the Glc*N*Ac and Mur*N*Ac residues by peptidoglycan deacetylases (PDAs). *N*-deacetylation is responsible for *B. anthracis* and *B. cereus* resistance to lysozyme. There is an unusual occurrence of 10 highly homologous PDAs in *B. anthracis* and *B. cereus* [57]. Among the PDAs of *B. anthracis*, three peptidoglycan GlcNAc deacetylases (PGNGdacs) have been identified, namely BA1977, BA1961, and BA3679. BA1977 is a bona fide peptidoglycan deacetylase involved in resistance to host lysozyme and required for full virulence [58]. BA1961 participates in the biogenesis of PG during both cell elongation and division, while Δ*ba1961* mutant strains shows a defect in cell separation and local thickenings of PG mainly at the septa [58]. Those PGNGdacs represent a validated antibiotic target (Figure 1B). Balomenou et al. demonstrated the in vitro effect of hydroxamate ligand *N*-hydroxy-4-(naphthalene-1-yl) benzamide (NHNB), a selective inhibitor of histone deacetylases-8, against two PGNGdacs, namely BC1974 and BC1960 from *B. cereus*, highly homologous to BA1977 (97%) and BA1961 (95%) of *B. anthracis*, respectively [59]. NHNB showed bactericidal activity against *B. cereus* and *B. anthracis*. However, the *B. anthracis* strain used in the study, *B. anthracis* 7702, is an uncapsulated strain; the effect of PGNGdacs inhibitors needs to be demonstrated on the *B. anthracis* capsulated strain.

*B. anthracis* PG is the target of β-lactam antibiotics such as penicillin G or amoxicillin, but those antibiotics are not specific to *B. anthracis* and intrinsic or inducible resistance to β-lactam antibiotics has been reported in clinical isolates [60,61,62]. Recently, in the search for antimicrobial molecules targeting *B. anthracis*, the interferon-inducible Glu-Leu-Arg-negative CXC chemokine CXCL10 was identified [63]. This molecule targets the FtsE/X, a complex that has been shown to be involved in activating cell-wall hydrolases that impact peptidoglycan remodeling during cellular elongation [64]. FtsE/X-dependent killing of vegetative cells of *B. anthracis* results from a loss of cell-wall integrity due to the disruption of PG processing (Figure 1B) [65].

PG is the target of PG hydrolases, a wide group of enzymes that catalyze the degradation of bacterial cell walls. These enzymes consist of muramidases, amidases, endopeptidases, carboxypeptidases, and glycosidases that serve as bacteriolysins, autolysins, and bacteriophage endolysins all with the ability to degrade selective cell-wall PG components. One promising approach for inactivation of *B. anthracis* is the use of bacteriophage endolysins or lytic enzymes encoded by bacterial genomes (autolysins) with highly evolved specificity toward bacterium-specific PG cell walls (see phage box).

### 3.1. Cell-Wall-Associated Glycopolymers

In Gram-positive organisms, the sacculus is densely functionalized with glycopolymers that are covalently bound to peptidoglycans. Such functionalization is important for survival. LytR-CpsA-Psr (LCP) enzymes are MPs that have been implicated in attaching WTA to peptidoglycan in *B. subtilis* and *S. aureus.* LCP enzymes form a phosphodiester bond between O6 of *N*-acetylmuramic acid (MurNAc) in glycan strands and O1 of GlcNAc in murein linkage units [66,67] as well as capsular polysaccharides (CPS) of *S. aureus* and several streptococcal species [68,69]. However, no WTA is synthetized by *B. anthracis* [24], as the bacterium lacks the genes for ribitol phosphate (RboP) teichoic acid synthesis. *B. anthracis* produces a PDGA capsule, which is tethered via amide bonds to meso-DAP of PG [27,70]. The bacterium possesses six different genes encoding LCP enzymes. Liszewski Zilla and co-workers showed that SCWP synthesis in *B. anthracis* requires the LCP family of enzymes [71,72]. Presumably, LCP enzymes transfer polymerized SCWP repeats from undecaprenol onto PG. Typically, LCP proteins encompass a short *N*-terminal cytoplasmic domain, followed by 1 to 5 transmembrane helices and a C-terminal LCP domain of approximately 150 residues, which is thought to be exposed on the trans side of the membrane [73]. The inhibition of LCP enzymes could be a good therapeutic target to tackle bacterial infections, as the target enzymes are implicated in the assembly of many bacterial cell-wall polymers. In order to guarantee a stringent spectrum of action of such inhibiting compounds, the structure determination of these enzymes for each pathogen of interest should be accomplished. Unveiling the atomic structure of the *B. anthracis* LCP enzymes in complex with their substrates, for example, would provide precious insights that could structure-guide the design of specific LCP inhibitors (Figure 1B).

### 3.2. Secondary Cell-Wall Polysaccharide (SCWP)

**Figure 2:**
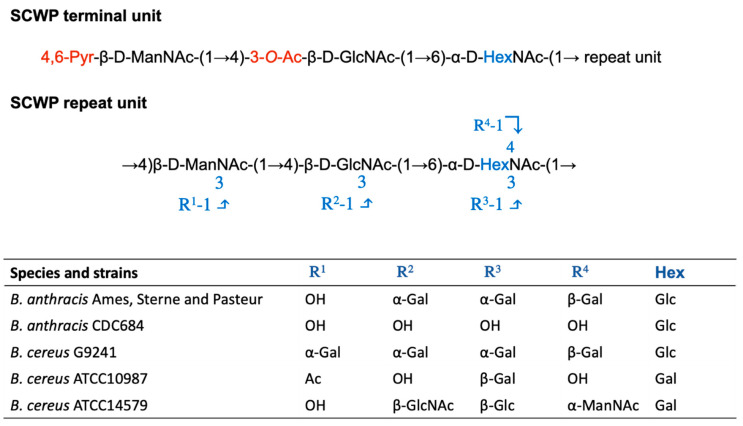
Structural variation of the SCWP trisaccharide units amongst various members of the *Bacillus cereus* group. Structure of the terminal and the repeat units of *B. cereus* sensu lato SCWP. In black is the common trisaccharide backbone, in blue the variation as indicated in the table for *B. anthracis*, *B. cereus* G9241, *B. cereus* ATCC 10987, and *B. cereus* ATCC 14579.

The SCWP is covalently attached to PG through an acid-labile phosphodiester bond. It is essential for cell growth, shape, division, and it anchors surface layer proteins (SLPs) to the cell wall. The SCWP of *B. anthracis* is a polysaccharide with the repeating structure [→4)-β-ManNAc-(1→4)-β-GlcNAc-(1→6)-α-GlcNAc-(1→]*n*, where α-GlcNAc is replaced with α-Gal and β-Gal at O-3 and O-4, respectively, and the β-GlcNAc is replaced with α-Gal at O-3 [74] (Figure 2). *B. cereus* G9241 and other *B. cereus* isolates, causing anthrax-like disease, synthesize SCWP with similar size and structure as *B. anthracis*, although wall polysaccharide from these isolates carries an additional α-Gal substitution at O3 of ManNAc [75].

The repeating unit of the SCWP main chain of *B. anthracis* and *B. cereus* appears to be similar except that α-GlcNAc is replaced by GalNAc [76]. Strain-dependent SCWP decoration may occur at O3 and O4 of the α-HexNAc, and at O3 of the β-GlcNAc residues resulting in considerable complexity in the polymer structure (Figure 2) [76,77]. Despite these variations, it has been established that the SCWPs of the *B. cereus* group contain a conserved unit that caps the non-reducing end of the polymer that contains specialized modifications. The terminal unit contains a ketal pyruvyl group occurring at both the C4 and C6 hydroxyl groups of the terminal ManNAc residue, an O-acetyl group at the C3 hydroxyl, and the α-GlcNAc is de-*N*-acetylated [78]. Non-covalent interactions with SCWP represent the anchoring strategy to the cell wall for many proteins, such as SLPs, to the cell wall (see S-layer anchoring section).

The *B. anthracis* SCWP is essential and plays a key role in the organization of the cell envelope of vegetative cells, and it is intimately involved in host–pathogen interactions. Targeting the SCWP component or the enzymes responsible for its synthesis could be an interesting way of combatting anthrax, especially since *B. anthracis* SCWP shows some differences with the one in *B. cereus* (Figure 1B). Moreover, SCWP galactosylation appears to be important for virulence, since *B. anthracis* CDC684 lacking all SCWP galactosyl modifications is avirulent in a mouse model of infection [79]. In addition to that, a *B. anthracis* Ames mutant lacking *galE1*, a UDP-glucose 4-epimerase, required for SCWP galactosylation exhibits reduced encapsulation and decreased virulence in a murine model of anthrax [80]. The enzymes required for incorporation of β- and α-Gal in *B. anthracis* SCWP are specific of *B. anthracis* and *B. cereus anthracis* such as G9241 [81]. Furthermore, the enzymes necessary for α-Gal incorporation in *B. anthracis* SCWP appear essential, unless either *galE1* or *gtsE*, the gene encoding for the enzyme responsible for β-Gal incorporation in *B. anthracis* SCWP, is deleted. The specific pattern of SCWP galactosylation is used for detection of *B. anthracis*. The monoclonal antibody EAII6G6 [82,83], used diagnostically to identify *B. anthracis* strains, recognizes a cell-wall polysaccharide epitope, with particular emphasis on the presentation and arrangement of Gal residues carried on the HexNAc backbone, specific for and expressed by *B. anthracis* strains except the CDC686 strain (lacking all SCWP galactosyl modifications) and with low specificity to *B. cereus* G9241 (Figure 1B) [84].

The SCWP is a ligand for murein hydrolases of bacteriophages, for example PlyG and PlyL [85,86]. PlyG, which is encoded by the γ-phage, binds to the SCWP from *B. anthracis* and *B. cereus* G9241, but not to the wall polysaccharides of *B. cereus* ATCC 10987 and ATCC 14597, the reference strains in the *B. cereus sensu stricto* group [85,87]. The absence of galactose in the SCWP of *galE1* mutant strain decrease PlyG binding to *B. anthracis* [80]. SCWP galactosylation is required for the proper functioning of PlyG and PlyL murein hydrolases, which are described to be dependent on the β-Gal modification at O4 of α-GlcNAc in the repeating trisaccharide [88]. In fact, the affinity of PlyG for a chemically synthetized trisaccharide backbone with a β-Gal in O4 of α-GlcNAc is high compared to the affinity of PlyG for a chemically synthetized trisaccharide backbone with all the substitutions (as observed in SCWP) [88]. However, the absence of β-Gal modification in the SCWP of *B. anthracis* strain lacking GtsE, the enzyme required for β-Gal incorporation, increases the binding of PlyG to the SCWP suggesting that β-Gal modification may hinder PlyG binding or that PlyG preferentially bind repeats with α-Gal modification in the SCWP [81]. Thus, the SCWP of *B. anthracis* is specifically targeted by the PlyG endolysin. A quantitative analysis of the thermal stability of PlyG has proven the thermostability of this enzyme, a characteristic that increases the general interest in its potential use as an antibacterial agent, as it shows a prolonged therapeutic shelf life expectancy [89].

### 3.3. Peptidoglycan Covalently Anchored Proteins

In Gram-positive bacteria, covalent anchoring of surface proteins to the peptidoglycan is orchestrated by sortases (Srts). Srts link proteins harboring a C-terminal LPXTG sorting signal (L = leucine, P = proline, X = any amino acid, T = threonine, G = glycine) at their C-terminus to the PG. This results in a covalent link with the PG through transpeptidation, creating a new peptide bond between the C-terminus of the recognition sequence and the cell wall. Based on its genome sequence, *B. anthracis* appears to have nine to 11 LPXTG-containing proteins (or just sortase targets) depending on the analyzed strain [30,31,90]. The function of some of these LPXTG proteins has been described. BasB and BasC, anchored by SrtA [30,31], are, for example, adhesins that bind in a dose-dependent manner to bovine type I collagen and promote attachment of the microorganism to collagen when expressed in a heterologous bacterium [91]. GamR, also anchored by SrtA, is used by the *γ* phage to adhere to the bacillus surface [92]. IsdC, anchored by SrtB, is an haem-iron acceptor [35]. BasI and BasH are MPs anchored to the PG of the sporulating bacillus by SrtC [39]. As mentioned earlier, *B. anthracis srtA-srtB-srtC* mutant is as virulent as the parental strain, suggesting that none of the LPXTG proteins is essential for virulence. However, as they are surface exposed proteins, they can be a good vaccine candidate. Among the *B. anthracis* immunogenic secreted proteins identified by Gat et al. several LPXTG were identified [93].

## 4. The Proteinaceous Armor: The S-layer

For a myriad of bacteria and nearly all archaea, the outermost cell surface component consists of a regular semipermeable (glyco)protein monolayer, known as the surface layer or S-layer [94,95]. Upon secretion to the cell surface, S-layer proteins (SLPs) engage with cell surface polymers and self-assemble into a paracrystalline 2D lattice with defined symmetry. Though S-layer envelope components are widespread across prokaryotes, their structure and function are still poorly understood. In archaea, the S-layer is often implicated in maintaining the cell shape, as they lack other cell-wall features [94].

**Figure 3:**
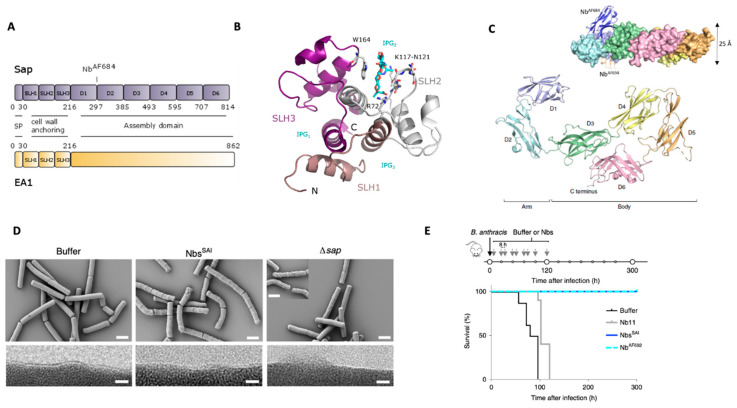
S-layer architecture of *B. anthracis.* (**A**) Domain architecture of Sap and EA1 S-layer proteins. The *N*-terminal ~216 residues comprise a signal peptide (SP) and a pseudo-repeat of three SLH motifs that form a cell-wall-anchoring domain. Sap assembly domain comprises of 6 independent domains as demonstrated by Sap^AD^ crystal structure reported in Fioravanti et al., 2019 (PDB: 6HHU [19]). The site where the therapeutic nanobody AF684 is binding as in upper C panel is indicated (PDB: 6QX4 [19]). (**B**) Crystal structure of the cell-wall anchoring domain of Sap in complex with a synthetic SCWP unit (IPG). Each of the 3 SLH motifs are highlighted forming a pseudo-trimer shaping 3 SCWP-binding grooves (IPG1-3). Residues important in SCWP binding are highlighted. The S-layer is oriented away from the image plane (C-terminus) with the *N*-terminus and terminal SCWP unit facing toward us (PDB: 6BT4 adapted from [56]. (**C**) X-ray structure of Sap^AD^ (residues 216–814) of Sap showing an arm-and-body organization composed of 6 β-sandwich domains. The inset shows the nanobodies used to solve the crystal structure. Notable Nb^AF684^, which is binding the hinge region displays therapeutic activity. (**D**) Scanning and transmission electron microscopy (SEM and TEM, top and bottom row, respectively) representing the morphology defect of *B. anthracis* 34F2 cells treated with buffer or Sap assembly inhibitory nanobodies (Nbs^SAI^), as well as RBA91 cells (Δ*sap*) treated with buffer. RBA91 and cells treated with Nbs^SAI^ present a scoured phenotype. TEM images show the loss of the ordered surface monolayer in RBA91 and in Nbs^SAI^-treated cells. Scale bars, 2 μm and 10 nm for SEM and TEM images, respectively (adapted from Fioravanti et al., 2019). (**E**) Schematic of the treatment regime and survival curves (bottom) of *B. anthracis* infection and Nb treatment studies in mice. The treatment consisted of a 6 days course of 10 subcutaneous 100 μL doses of 200 μM Nbs or buffer after infection. Mice receiving buffer or Nb11 (a non-related S-layer Nbs) injections would succumb to lethal anthrax within ~110 h post infection; mice receiving Nbs^SAI^ or Nb^AF692^ (the most potent Nbs^SAI^) treatment are able to recover from the infection and survive anthrax disease. *n* = 8 mice per group. From Fioravanti et al., 2019.

In bacteria, on the other hand, the S-layer is reported to exploit multiple functions ranging from a protective and selective coat to an adhesive surface [95,96]. Nevertheless, experimentally verified structure–function correlations are far and between. The *B. anthracis* S-layer was first observed in 1969 as a smooth crystalline surface through the use of freeze–fracture electron microscopy (Holt and Leadbetter 1969). However, only in the 1990s, both S-layer proteins (SLPs) were identified. Interestingly, this organism has two S-layers that are mutually exclusive and interchange as a function of its life cycle [97]. The surface array protein (Sap) is the main S-layer component associated with the bacterium’s exponential phase of growth. Sap gets gradually displaced by the extractable antigen 1 (EA1), which is the main SLP forming the stationary phase S-layer. This developmental switch is controlled by phase-specific transcription factors, and in addition, *eag* transcription is possibly repressed by both the Sap and EA1 proteins [97]. Both proteins are synthesized as proproteins and build up in typical fashion for a Gram-positive SLP: an *N*-terminal secretion signal, a tripartite SLH-motif for cell-wall anchoring, and the C-terminal assembly domain (Figure 3A). The *N*-terminal peptide is cleaved upon secretion by the S-layer dedicated accessory SEC system [98]. The two proteins share high similarity in their SLH-domains for cell-wall anchoring (74%) but only limited sequence similarity (42%) and identity (22%) in their assembly domains. For Gram-positive bacteria, the SLH-domain is a common motive found in the cell-wall anchoring domain of proteins that are localized at the bacterium surface [99], such as SLPs and S-layer-associated proteins (see BSLs section). The exclusively tripartite domain forms a three-pronged spindle with a central three-helix bundle (Figure 3B [100]). Conserved anionic and cationic residues in the grooves between the spokes form the non-covalent interaction interface with the terminal PG-anchored SCWP unit [56,101]. Recently, atomic resolution insights in the assembly domain of Sap (Sap^AD^) were obtained [19]. By nanobody-assisted X-ray crystallography, the structure of Sap^AD^ could be resolved to 2.7 Å resolution, representing only the second complete S-layer assembly domain ever solved for a Gram-positive bacterium [102] and the first belonging to a pathogen. Sap^AD^ is an extensive multi-domain protein that presents six β-sandwich domains connected by short linkers (Figure 3A,C). In solution, it adopts a flat tile-like supertertiary structure consisting of an “arm” (domain 1 and 2) and “body” (domain 3 to 6) (Figure 3C [19]). The lattice topology of the Sap and EA1 SLPs has been studied extensively with electron diffraction. Negative staining of isolated S-layer fragments of deflated cells revealed the lattice parameters for both Sap and EA1 [103]. Both Sap and EA1 maps were reconstructed using *p1*-symmetry and calculated to 20 Å and 30 Å for EA1 and Sap, respectively. The unit cell parameters derived from the projection maps were a = 69 Å, b = 83 Å, and γ = 106° for EA1 and a = 184 Å, b = 81 Å, and γ = 84° for the Sap array. More recently, in vitro re-crystallized S-layer projection maps were calculated for EA1 [104] and Sap [19]. While the resolution and unit cell parameters were comparable for EA1, the new Sap diffraction map showed a slightly larger unit cell of a = 211.4 ± 1.9 Å, b = 89.1 ± 0.7 Å, and γ = 84.0 ± 1.0°, an about 10% size discrepancy possibly due to the different behavior of the S-layers in negative stain and electron cryo-microscopy. The latter map was of higher detail and clearly showed the *p2*-symmetry of the Sap maps with a cross-and-ridge-like lattice architecture [19]. These observations suggest that both re-crystallized Sap and EA1 S-layers at least partially reflect the subunit packing on top of the cells. In contrast to other S-layers observed in nature [95,105], Sap is also forming higher-molecular-weight species in the absence of divalent cations [19], but EA1 is still dependent on cations for its polymerization (unpublished results). Interestingly, as observed by freeze–etch and negative stain electron microscopy, the Sap and EA1 S-layer seem to have different “settings” on the cell [103]. Whereas the Sap S-layer is more continuous, the EA1 S-layer is patchier and features more breakages when assessed on cell, possibly indicating that Sap is more flexible and thus adapted to cover fast-dividing cells in the exponential phase. The Sap–EA1 distribution is likely important in the positioning of some S-layer-associated proteins (BSLs), and it has been shown that the Sap-EA1 interface is important in BslO protein deposition at nascent cell division sites, thus directly influencing cell division [51]. BslO is in fact a BSL protein with *N*-acetylglucosaminidase activity that catalyzes mother–daughter cell separation [106]. Other functions of the S-layer are less understood. Our recent study targeting the Sap S-layer through the use of nanobodies (Nbs) as bio-tools raises, however, some interesting questions [19]. In this study, the link between SLP presence and virulence was investigated. We identified anti Sap Nbs able to prevent Sap polymerization that can also depolymerize pre-formed Sap S-layer in vitro. When applied in vivo, these anti Sap S-layer inhibitory nanobodies (Nb^SAI^) induced severe morphological and growth defects (Figure 3D). Nbs^SAI^ treated cells would first shrivel and then collapse. The Nb-induced defects [19] are more outspoken compared to the defect observed for the *sap*-deletion mutant RBA91 (Figure 3D) [97,107]. This might be due to the fact that Nbs induce an acute loss of S-layer leading to more drastic effects on the cell envelope when compared to the *sap* genetic knockout. Cells with a genetic knockout of *sap* would have more time to adapt and shift to an EA1 S-layer that seems to be able to rescue these defects. These results suggest that the S-layer is a more important cell shape determinant than previously anticipated. Interestingly, the nanobodies were shown to offer a protective effect in vivo in an anthrax mouse model of infection, suggesting that also the virulence of *B. anthracis* is impaired (Figure 3E). The link between virulence and S-layer integrity is less outspoken than for the capsule and toxin genes, but this new observation does point to the S-layer as a proper virulence factor and a promising therapeutic target (Figure 1B). As such, immunization with EA1 was previously found to offer a protective effect in a mouse model of inhalational anthrax [18]. Important to note here is that it is poorly understood at which point the capsule is present during the infection process implying that the S-layer can indeed form the interface with the environment at least in some stages of the infection and the bacterium’s life cycle. This hypothesis is reinforced also by the fact that Sap is found to be a target for bacteriophage infection of vegetative cells [108] that llamas immunized with spores are found to produce antibodies specific for EA1, but most importantly, that both Sap and EA1 have been shown to be immunogenic in the course of human anthrax [109].

Finally, it is important to notice that EA1 has been found in *B. anthracis* spore preparation, and it could be part of the spore’s coat [110,111]. Its role in the spore’s coat is yet unknown, and Williams and co-workers have proposed that EA1 presence in spore preparation could be simply a persistent contaminant of spores preparation, an artefact due to lab procedure [112]. However, in 2008, Love and co-workers, through competitive panning, obtained highly specific single chain antibodies (scFvs) against EA1, which are capable of detecting *B. anthracis* in vitro with almost no cross-reactivity of proteins from related species (Figure 1B) [113]. Since then, EA1 has become a valuable biomarker for the detection of both vegetative and dormant *B. anthracis* cells [113].

### 4.1. Surface-Localized Proteins

In addition to Sap and EA1, the chromosome of *B. anthracis* holds 19 genes encoding SLH harboring proteins; these gene products have been designated as *Bacillus* S-layer-associated proteins (BSLs) [114]. Some of them have been studied as potential vaccine candidates. Proteins with SLH domains are found both on the surface and in the secreted fraction of *B. anthracis*. The immunodominant SLH domain-containing proteins are prominent targets in anthrax vaccine development to generate antibody response against the bacterium [115,116].

The *bslK* gene product carries a NEAT (heme-binding near-iron transporter) domain and binds heme-iron [117]. A vaccine formulation consisting of recombinant proteins from a surface-localized heme transport system containing NEAT domains was attempted, and its efficacy was investigated [118,119]. A cocktail of five NEAT domains, including the one of BslK, was found to provide protection against a lethal challenge of *B. anthracis* spores (subcutaneously injected or inhaled) [118,119]. In the case of lethal challenge with inhaled *B. anthracis* spores, reduction in the formulation to three NEATs (IsdX1, IsdX2 and Bslk) was as effective as a five NEAT domain cocktail [119]. These results support the further development of recombinant heme transporters as a potentially effective vaccine preventing anthrax disease (Figure 1B).

BA3338 is an immunodominant SLH domain carrying protein. BA3338 is highly similar to internalin, a surface protein that is anchored to the cell wall of several pathogens and promotes the bacterial entry into host cells. [116,120]. Recently, Kumar et al. showed that an improvement of PA vaccine protection could be obtained if mice were challenged with PA in combination with BA3338. Therefore, the BA3338 represent a co-vaccine candidate to augment protection efficacy for development of the next-generation PA-based subunit vaccine [121]. This study established that the BA3338 stimulate humoral and Th2 type immune responses for effective opsonophagocytosis, and it is able to enhance protective efficacy against *B. anthracis.*

BslA, a pXO1-encoded adhesin [114], adheres to mammalian cells and functions as a competitive inhibitor for the binding of bacilli [98]. In a mouse model for disseminated anthrax meningitis, *bslA* mutant bacilli were defective in crossing the blood–brain barrier and in establishing anthrax [122]. Thus, BslA represents an adhesin and virulence factor for *B. anthracis*. Deletion of the *bslA* gene fully attenuated a Vollum ∆pXO2 strain in intravenous inoculation of rabbits and prevents central nervous system infections, possibly leading to the generation of a safer vaccine [123].

### 4.2. S-layer Anchoring

Biochemical studies have shown that the S-layer homology (SLH) domain of the S-layer and S-layer-associated proteins (SLPs and BSLs, respectively) of *B. anthracis* is anchored to the SCWP through noncovalent interactions [124]. Structural examinations of SCWP of *B. anthracis* revealed that the distal trisaccharide is modified by a 4,6-*O*-pyruvyl ketal on the β-ManNAc residue [74,75] (Figure 2). Genetic studies demonstrated that *csaB*, which is the gene responsible for pyruvylation, is critical for retention of SLH-containing proteins [125]. Each of the three putative SCWP binding sites of the SLH domain contains a conserved basic residue (lysine or arginine) that is predicted to be involved in binding the negatively charged pyruvate ketal [100]. The X-ray crystal structure of the SLH domain of Sap in complex with 4,6-O-ketal-pyruvyl-β-ManNAc-(1,4)-β-GlcNAc-(1,6)-α-GlcN reveals that the conserved terminal SCWP unit is the direct ligand for the SLH domain and the binding interactions identified account for the requirement of 4,6-O-ketal-pyruvyl-ManNAc (Figure 3B) [56]. Furthermore, direct binding studies between a synthetic single pyruvylated ManNAc monosaccharide and S-layer or S-layer-associated protein recently confirmed this interaction [126].

The C-3 hydroxyl of the β-GlcNAc moiety of the distal repeating unit is non-stoichiometrically modified by an acetyl ester (Figure 2), and the α-GalNAc residue can be *N*-deacetylated [78]. A model for *O*-acetylation of SCWP has been proposed in which the membrane-bound *O*-acetyltransferases *patB1* performs the acetylation, whereas *patB2* is required for translocation of an acetyl donor across the plasma membrane from a cytoplasmic source [127]. Genetic studies have indicated that strain deleted of *patA1* and *patA2*, which significantly reduces O-acetylation of SWCP, assembles an S-layer that cannot retain the S-layer protein EA1, and is mainly composed of Sap [128]. It was also observed that two SLH-containing proteins such as BslO and BslA, which facilitates infection by mediating adherence to host cells, were not retained at the cell surface and released in the medium. These data suggested that SCWP O-acetylation mediates two different and important determinants of *B. anthracis* virulence: chain length regulation to evade phagocytosis and adherence to the host tissues. However, the X-ray crystal structure of the SLH domain of Sap in complex with 4,6-O-ketal-pyruvyl-β-ManNAc-(1,4)-β-GlcNAc-(1,6)-α-GlcN reveals the insignificance of the O-acetylation on the GlcNAc residue for recognition by Sap [56]. In addition, recent in vitro direct binding studies with synthetic trisaccharides and monosaccharides derived from the distal unit of *B. anthracis* SCWP that have perturbations in the 4,6-O-pyruvyl ketal and O- and *N*-acetylation patterns showed that O-acetylation of the GlcNAc moiety did not substantially impact association with S-layer and several SLH containing proteins [126]. However, the binding studies have been performed with individual proteins and do not probe possible retention of proteins through protein−protein complexes. In this respect, it may be possible that deposition of one protein that requires O-acetylated SCWP mediates the retention of other proteins. To date, the role of the de-*N*-acetylation of α-GlcNAc is not known.

Phage Box: ***B. anthracis*** **specific phages as detection, decontamination, or therapeutic agents**.

Lytic bacteriophages (phages) possess the ability to specifically recognize cell surface-localized receptors and infect and lyse a particular host. Phage strain specificity has been exploited for many decades as a means of uniquely identifying target bacteria, the so-called phage typing, through the use of phages or phage-derived products. In case of a *B. anthracis* outbreak or an intentional release of spores during an anthrax terrorist attack, a rapid and unequivocal method for detection is required. The differentiation of *B. anthracis* from strains belonging to the *cereus* group has been proven challenging, both with the use of standard microbiology methods and methods depending on antigen recognition for *B. anthracis* detection [129]. To date, DNA-based identification methods are the most reliable detection methods, but they cannot discriminate between alive and dead bacilli [130]. Phages may have the potential to be used in *B. anthracis* detection in clinical and environmental samples. Moreover, they could be used in bioremediation of contaminated areas and as antimicrobial treatment [118,131]. *B. anthracis* spores can persist in the environment in hostile condition for decades, and in addition to that, large areas can be impacted by anthrax contamination as spores can be aerosolized. Water and a broad range of food products can be contaminated by anthrax spores, representing a real treat in the raise of fatal gastrointestinal anthrax as spores are renowned for surviving the cooking of contaminated food. In this scenario, a novel approach for decontamination that employs phages would represent a cheap and environmental/human-friendly alternative to caustic chemical solutions or other physical methods currently used such as gamma irradiation, ultraviolet light, and high pressure [132,133]. Phages need viable vegetative forms of bacteria for their reproductive cycle; therefore, using phages to decontaminate a certain area of *B. anthracis* spores is problematic. To circumvent this problem, contaminated areas should be exposed to germinating agents before phage treatment. There are, however, few filamentous phages that specifically bind to *B. anthracis* spores [134,135,136,137], but since these do not interact with the cell envelope, the components will not be discussed here.

Phages active against *B. anthracis* need to encode capsule depolymerases able to degrade the PDGA capsule that may be present at the bacterial surface [138]. Gamma-phage susceptibility is a standard test that has been extensively used for routine identification of *B. anthracis* and for its discrimination from closely related *B. cereus* group species (Figure 1B) [139,140]. A first drawback of this method is that gamma phage is unable to lyse capsulated *B. anthracis* [141]. This will impair the access of the phage to its receptor, the GamR PG-anchored protein [92]. Although gamma phage is highly specific for *B. anthracis*, there are a few *B. cereus* strains that can be infected by this phage [92]. Therefore, the World Health Organization does not suggest the use of Gamma phage as a sole means for *B. anthracis* identification and detection, but instead, it can be used in conjunction with other tests [129]. A faster (12 h versus 4/5 days) and more sensitive phage typing can be performed with the Wp1 phage [142]. The narrow host range of this phage and the identification of the phage protein p23 involved in such recognition have opened an opportunity to develop a detection method based on the use of a recombinant receptor-binding protein. Another promising *B. anthracis*-specific phage is the AP50 phage [143,144]. It exhibits a narrow host range, infecting *B. anthracis* exclusively, although one *B. thuringiensis* strain (ATCC 35646) was shown to possess an AP50-like element. Identification of spontaneous bacterial mutants resistant to the AP50c killing were found to have mutations in the *csaB* gene [145], which encodes for a protein involved in cell surface anchoring of S-layer and S-layer-associated proteins (see S-layer anchoring section) [125]. In the same study, the S-layer protein Sap was identified as the binding receptor of this phage. Nevertheless, for this phage, a deeper investigation needs to be performed in order to clarify its potential in phage typing or phage therapy and remediation; it is not clear in fact if AP50c or its receptor-binding protein can reach the Sap S-layer of a capsulated bacilli.

*B. anthracis* is usually a drug-sensitive strain (penicillin G, amoxicillin or ciprofloxacin), but anthrax prognosis depends on the time after which the pathogen is identified and the application of appropriated therapy. A study conducted by Cavallo and co-workers on *B. anthracis* isolates from humans, animals, and environmental samples revealed that 11% of the strains were naturally resistant to antibiotics [146], indicating a potential rise in resistant bacilli as a result of long-term exposure to antibiotic treatment. In addition to that, multidrug-resistant strains could be deliberately engineered and released during a terrorist attack. In this context, it is interesting to note that the bacteria’s resistance to antibiotics does not contribute to the formation of phage resistance, thus phages may work on antibiotic-resistant *B. anthracis* strains. As a caveat, many phages specific to *Bacillus* species mediate generalized transduction [147], which, during phage therapy, could result in the transfer of anthrax toxin genes (so-called phage conversion) [148], and this occurs via temperate phage-mediated gene transfer between species [149]. A cocktail of different phages could be useful to narrow down the host range recognition of phages even more, as well as avoiding the possible development of bacterial phage resistance. Phage derivatives on the other hand would be the safest bio-tool to use for typing, decontamination, and therapy in addition to the fact that their size allows them to reach the bacterial surface in the presence of the capsule. To date, several endolysins have been characterized showing a great application potential. A promising example of such phage derivatives is represented by endolysins: phage enzymes that specifically lysing the bacterial PG cell wall and that present high strain specificity in addition to the capability of lyse the bacteria in a matter of seconds/minutes [150]. PlyG, the endolysin isolated from the Gamma phage, has been presented as a promising bio-tool to be used against *B. anthracis*. Schuch and coworkers have shown that if the bacteria can become resistant to gamma-phages, they still remain sensitive to PlyG [85]. The PlyB lysin has been described to be lytic in *B. anthracis*-like strains [151,152,153]. Another promising bacteriolytic enzyme PlyPH was shown to be specific for *B. anthracis* Sterne strain and *B. cereus* strain RSVF1 (this last strain is a representative of *B. anthracis* cured of its virulence plasmids) [154]. PlyPH remains active between pH 4 and 10.5, and a single dose rescued 40% of mice infected intraperitoneally with an attenuated *B. anthracis* strain. Other endolysin-targeting *B. anthracis* have been described and some, such as PlyG, bind to secondary cell-wall polysaccharides (SCWP). The combination of multiple lysins that show different host-dependent lytic activities and cleave different PG bonds could be exploited as therapeutics or decontamination strategy. One last example of a promising phage-derived bio-tool to be exploited in the anthrax field is the prophage-derived autolysin (AmiBA2446) from *B. anthracis,* which activity was demonstrated significant against both *B. anthracis* and *B. cereus* strains [155]. PG hydrolases have a modular architecture consisting of one or several catalytic domains, which break down bacterial cell-wall components and cell-wall binding domains (CBDs) that recognize a highly specific ligand in the PG. Such high specificity can be exploited in both bacterial species detection and selective killing. Recently, Kim et al. showed that silver nanoparticles, suitably conjugated to the AmiBA2446-CBD, can efficiently target and selectively kill *B. anthracis* over *Bacillus subtilis* and *Staphylococcus aureus* [156]. Therefore, this new biologically assisted hybrid strategy has the potential to provide a selective decontamination strategy of pathogenic bacteria with minimal impact on normal microflora.

## 5. The Invisibility Cloak: The Capsule

The outermost component of a fully virulent *B. anthracis* cell envelope is a poly-γ-D-glutamic acid capsule (PDGA) (Figure 1). This capsule appears exterior to the S-layer of vegetative cells, threading through the 2D crystal pores. Apart from exotoxins, that critically suppress the host immune system [157,158], the *B. anthracis* capsule represents the other essential virulent factor [159]. These virulence factors are plasmid-associated. In particular, it is the pXO1 and pXO2 plasmids that harbor the anthrax toxins and the capsule biosynthetic genes operons, respectively [160,161]. Numerous studies have highlighted the key role played by PDGA capsule in anthrax pathogenesis, suggesting its potential as a potent target for the development of novel intervention strategies to counter anthrax infections. The capsule is synthetized by the three membrane-associated enzymes (CapA, B, and C) that are encoded by the *capBCAD* operon located on pXO2 [162]. It has been shown that the capsule expression is activated and modulated by a choreography of mechanisms that take place in response to environmental signals, such as CO_2_ and bicarbonate fluctuations, as well as transcription factors that are encoded from both virulence plasmids.

The *acpA* and *apcB* pXO2 genes were described to encode for transcription factors that positively control, in a bicarbonate-mediated fashion, the *cap* operon [163,164,165]. In a mouse model for inhalational anthrax, the virulence of a strain carrying both virulence plasmids but deleted specifically for *capBCAD* was reported to be highly attenuated. In particular, mutant spores could germinate in the lungs but not disseminate to the spleen [159]. A strain preserving the *cap* operon but possessing a mutated *acpB* gene, displayed an elevated LD_50_ and reduced ability to disseminate [159].

In addition to the *acpA* and *apcB* pXO2 genes, from in vitro studies, it appeared that capsule synthesis was enhanced by the global regulator of virulence AtxA [165]. Encoded by *atxA* gene, which is located within the pathogenic island on pXO1, AtxA was reported to activate in vitro the transcription of both toxin and capsule genes in the presence of CO_2_ and bicarbonate [165]. A more recent study demonstrated that in vivo AtxA is not required for capsule biosynthesis as an *atxA* mutant strain was capsulated in vivo and reported to be as virulent as the WT strain (pXO1^+^, pXO2^+^) in an anthrax mouse model of infection [166].

The fourth gene of the *cap* operon encodes CapD, a gamma-glutamyltranspeptidase required for the covalent anchoring of polyglutamate to the peptidoglycan layer [70,167]. This enzyme cleaves poly-γ-D-glutamate capsule and generates amide bonds with peptidoglycan cross-bridges to attach the capsule into the envelope of *B. anthracis*. *capD* mutants have shown reduced virulence in various animal models of anthrax infection [27,167,168], and thus, the enzyme itself has been the object of several studies aiming to control its function. One of these studies has reported that Capsidin, a non-competitive inhibitor that specifically acetylates the active site threonine of CapD transpeptidase, could function as promising CapD inhibitor [70]. The anchoring of the PDGA capsule has also been found to be impaired in two peptidoglycan *N*-deacetylase mutants: the Ba1961 and Ba3679 [169]. These two enzymes appear to be involved in generating free amino groups by *N*-deacetylating the GlcNAc residues that create the site on the PG on which CapD anchors PGA. Impairing *N*-deacetylases activity would then constitute an interesting drug target, as simultaneously, the bacterium resistance to lysozyme as well as the resistance to macrophages phagocytosis could be affected (Figure 1B).

CapD is a bifunctional enzyme, as it also functions as a PDGA depolymerase, depolymerizing large capsule polymers and releasing lower-molecular-weight D-glutamic acid polymers into the environment [167,170]. Following this evidence, an effort to investigate the potential of CapD as a possible therapeutic tool to depolymerize the capsule during anthrax infection has been made (Figure 1B) [171,172]. Additional parenteral administration of CapD provided some protection to mice when co-injected with *B. anthracis* or provided 30 h post infection [171]. Later attempts to improve pharmaceutical stability of CapD led to a marked reduction in CapD capsule degrading capacity [173]. In 2014, Negus and Taylor performed a study that identified the *Pusillimonas noertemanii* EnvD enzyme. EnvD rapidly hydrolyses PGA and presents an increased stability at 37°C to that of CapD, interesting characteristics of a promising bio-tool to be taken into account to be investigated as novel therapeutic for the treatment of anthrax [172].

Similar to an invisibility cloak, the capsule hides surface antigens from the host immune system. A recent work from Sharma et al. clarified the role of the poly-γ- D-glutamate capsule in immune evasion of *B. anthracis* cells from macrophage-dependent opsonic phagocytosis. This study demonstrated that encapsulated virulent strain exhibit resistance toward complement-dependent and complement-independent bacterial phagocytosis by human macrophages. After incubation with normal human serum or heat-inactivated serum as well as serum-free media, the non-encapsulated strain became highly susceptible to phagocytosis by THP-1 macrophages [174]. In particular, an increased deposition of the derivate b of the complement component 3 (C3b) that serves as a potent opsonizing agent, was observed at the surface of cells lacking their capsule. By the use of complement pathway-specific component and component-deficient serum, the authors could also demonstrate that the classical pathway was primarily involved in mediating C3b binding on the non-encapsulated bacteria. In addition, an increase in the binding for other known mediators of complement fixation such as IgG, C-reactive protein, and serum amyloid P component was also observed on the non-encapsulated bacteria. Lastly, using a wide range of pHs, Sharma and co-workers could also demonstrate that the negative charge of the PDGA capsule is responsible for the differential binding of the complement proteins between the non-encapsulated and encapsulated strains [174].

The PDGA capsule alone is poorly immunogenic [175], but in the last few years, it has been shown that covalent conjugation or coupling of native capsule or synthetic peptides to various carrier proteins results in an improvement in anthrax protection [10,11,12,13,14,15,16,17]. Excellent results were obtained by Chabot and co-workers, who could demonstrate that covalently conjugating the PDGA to the outer membrane protein complex of *Neisseria meningitidis* serotype B leads to a complete protection of rhesus macaques against inhalational anthrax (Figure 1B) [176].

Finally, capsule staining is a gold standard microbiology method for the detection and identification of *B*. *anthracis* virulent cells. The capsule presence can be confirmed both by M’Fadyean staining, with polychrome methylene blue, or India ink staining (Figure 1B) [129]. M’Fadyean-stained cells will appear by light microscopy observation as blue-black bacilli surrounded by pink capsules, while with India ink, the capsule will correspond to a transparent halo around the cells. Despite the robustness of the discussed identification and detection methods, a huge drawback is these approaches require the culturing of the bacteria, a crucial and limiting step in emergency situations such as a terroristic attack. Recently, antibodies anti-PDGA capsule have been successfully used in a novel rapid multiplexed tests for the detection of different biological warfare agents directly from blood cultures [177].

## 6. Conclusions

Bacterial cell envelopes represent a crossroads of environmental/cell signals and responses, the interaction zone with the outside world and the battle ground where bacteria will defend themselves from hostile conditions.

*B. anthracis*, the etiological agent of anthrax, possesses a complex and dynamic composition of its cell envelope that changes in response to host/environmental signals. Despite the fact that anthrax nowadays is less of a public health problem in several developed countries, it remains a daily problem where zoonotic foci are present and a permanent worldwide threat as a bioweapon. Thus, it is mandatory to have improved methods and strategies for anthrax detection, decontamination, therapy, and vaccination that can be adopted in case of naturally occurring outbreaks or intentional release of this deadly bacterium.

In this review, we reported an overview of the progresses that has been made in the understanding of *B. anthracis* cell envelope components physiology and in their potential as identification, therapeutic, and vaccine targets. Among them is the promising recent report of in vivo data regarding the disruption of S-layer integrity as a novel anthrax therapeutic [19]. Excellent results in a primate model of inhalational anthrax infection were obtained for animals vaccinated with PDGA conjugated to the outer membrane protein complex of *Neisseria meningitidis* serotype B [176]. Finally, positive progresses were also made for *B. anthracis* detection and decontamination thanks to the use of specific *B. anthracis* phages or phages derivates that target cell envelope components [154,155,156].

These are few of the several encouraging results reviewed in this manuscript that were obtained from the study of *B. anthracis* cell envelope in the past decade. Despite the significant recent progress made since the last published review on this topic [16], many questions about the function and composition of cell envelope components remain unanswered, the understanding of which is critical to exploit them as full potential targets for the development of new strategies to face pending anthrax outbreaks.
